# Dual-Biomarker Oral-Rinse Testing for aMMP-8 and Calprotectin: A Potential Adjunctive Tool for Risk Stratification and Interdisciplinary Referral in Periodontitis and Type 2 Diabetes

**DOI:** 10.3390/bioengineering13091042

**Published:** 2026-09-08

**Authors:** Pietro Leone, Ismo T. Räisänen, Julie Toby Thomas, Pirjo Pärnänen, Sukumaran Anil, Andreas Grigoriadis, Dimitra Sakellari, Pierino di Silverio, Marco Evangelista, Timo Sorsa

**Affiliations:** 1 Smile Longevity Care 360 Centre, 80122 Naples, Italy; pietro_leone@hotmail.it; 2 Department of Oral and Maxillofacial Diseases, Head and Neck Center, University of Helsinki and Helsinki University Hospital, 00290 Helsinki, Finland; ismo.raisanen@helsinki.fi (I.T.R.); julie.tobythomas@helsinki.fi (J.T.T.);; 3 Center of Excellence in Precision Medicine and Digital Health, Department of Physiology, Faculty of Dentistry, Chulalongkorn University, Bangkok 10330, Thailand; drsanil@gmail.com; 4 Department of Periodontology and Implant Biology, School of Dentistry, Aristotle University of Thessaloniki, 54124 Thessaloniki, Greece; andreasgrigor@gmail.com (A.G.); dimisak@gmail.com (D.S.); 5 Dental Sector, 424 General Military Training Hospital, 56429 Thessaloniki, Greece; 6 Emerging Infectious Disease and High Contagiousness Unit, P.O. D. Cotugno-AORN Ospedali dei Colli, 80131 Naples, Italy; 7 Regional Observatory for Infectious Disease, 80100 Naples, Italy; 8 Azienda Ospedaliera dei Colli, Monaldi Hospital, Cardiovascular and Dysmetabolic Internal Medicine Unit, 80131 Naples, Italy; 9 Division of Oral Diseases, Department of Dental Medicine, Karolinska Institutet, 171 77 Stockholm, Sweden

**Keywords:** aMMP-8, calprotectin, point-of-care testing, oral rinse, periodontitis, type 2 diabetes, interdisciplinary referral, risk stratification, artificial intelligence

## Abstract

Periodontitis is a chronic, biofilm-induced inflammatory disease characterized by episodic connective tissue destruction and is epidemiologically associated with cardiometabolic conditions, notably type 2 diabetes (T2D) and cardiovascular disease (CVD). Conventional clinical signs do not reliably distinguish quiescent from actively progressing disease, which delays intervention and interdisciplinary referral. Chair-side point-of-care testing (POCT) of oral fluids offers a pragmatic, non-invasive adjunct for real-time assessment of disease activity, not only in dental but also in medical settings. This narrative review, reported in line with the Scale for the Assessment of Narrative Review Articles (SANRA), examines the clinical rationale and current evidence for oral-rinse POCT targeting active matrix metalloproteinase-8 (aMMP-8) and calprotectin. Oral-rinse aMMP-8 reflects disease-active collagenolysis and shows the more robust single-marker performance for periodontitis, whereas oral-fluid calprotectin, a neutrophil-derived S100A8/A9 protein, behaves as a predominantly local inflammatory signal and lacks validated oral-fluid reference ranges. Combining the two markers may add information beyond either alone when used in tandem as a complementary pair, but the supporting data are heterogeneous, largely exploratory, and hypothesis-generating. Dual-biomarker oral-rinse POCT is therefore best framed as a risk-stratification and referral aid rather than a stand-alone diagnostic, with combined interpretation potentially supported by AI technologies. Standardized pre-analytics, transparent threshold reporting, oral-fluid calprotectin reference values, and prospective multicenter validation are required before broad implementation.

## 1. Introduction

Periodontitis is a prevalent chronic inflammatory disease marked by episodic connective tissue breakdown that becomes irreversible when diagnosis and treatment are delayed [1,2,3]. In routine practice, clinical signs alone often fail to distinguish inactive disease from ongoing or future destructive activity, limiting the clinician’s ability to prioritize treatment intensity or identify patients who might benefit from broader medical evaluation. This limitation is clinically important because periodontitis shares inflammatory and metabolic pathways with type 2 diabetes (T2D) and cardiovascular disease (CVD) [4,5,6,7] and—beyond the cardiometabolic axis—with several other systemic conditions. Low-grade chronic inflammation, driven in part by neutrophil activation, oxidative stress, and dysregulated cytokine release, is now recognized as a contributor to obesity, T2D, atherosclerosis, and ischemic heart disease [4]. Within this paradigm, oral-fluid point-of-care testing (POCT) offers a practical way to operationalize biologically meaningful signals of disease activity during a routine dental visit.

Two oral-fluid analytes are of particular interest. Active matrix metalloproteinase-8 (aMMP-8, neutrophil collagenase-2) captures ongoing collagenolytic tissue breakdown [8,9]. Calprotectin (the S100A8/A9 heterodimer) reflects neutrophil-driven inflammatory burden and can modulate matrix metalloproteinase activity through zinc sequestration [10,11]. Because no single marker fully captures the multifactorial biology of periodontitis or its systemic links, a combined interpretation might help distinguish active from quiescent disease and flag patients for whom referral for dysglycemia or cardiovascular assessment could be appropriate. Such testing is intended to complement, not replace, conventional periodontal examination and clinical judgment [7,12,13].

This review focuses primarily on mouthrinse and oral-rinse applications of aMMP-8 and calprotectin in two settings: identifying periodontitis activity and monitoring the response to therapy, and supporting opportunistic, referral-oriented identification of individuals who may benefit from cardiometabolic risk assessment. Evidence from saliva and gingival crevicular fluid (GCF) is discussed where it informs biological plausibility, pre-analytics, or implementation. Diabetes screening can be conveniently performed during dental visits, which many individuals attend more regularly than medical appointments [14,15,16].

**What this review adds.** A recent special report by an overlapping author group proposed combining mouthrinse aMMP-8 and calprotectin with calcified carotid artery atheroma (CCAA) detection on panoramic radiographs as an integrated point-of-care approach [17], and earlier reviews have addressed aMMP-8 in the periodontitis–diabetes context [18]. The present review is narrower and more critical in scope: rather than advocating a combined test, it appraises where the evidence for combined interpretation is genuinely stronger than for aMMP-8 alone, where it is not, and what pre-analytical, threshold, and reference-range problems must be solved first. Its intended contribution is an honest, implementation-oriented synthesis of the pre-analytical realities, the cut-off controversy, and the asymmetric maturity of the two markers as monitoring tools, so that clinicians and researchers can judge the approach on its current evidentiary footing. This narrative review critically evaluates the clinical rationale and current evidence for dual-biomarker oral-rinse point-of-care testing of aMMP-8 and calprotectin as an adjunctive risk-stratification and interdisciplinary referral aid in periodontitis and T2D, and defines the pre-analytical, threshold-related, and validation requirements that must be met before clinical implementation.

## 2. Materials and Methods

This narrative review follows SANRA [19]. It is not a systematic or scoping review; we performed no formal screening, risk-of-bias appraisal, or quantitative synthesis, and the intent is thematic sufficiency rather than exhaustive inclusion.

We searched PubMed/MEDLINE (last searched July 2026) using combinations of controlled vocabulary and free-text terms for periodontitis, type 2 diabetes and dysglycemia, point-of-care and chair-side testing, oral rinse and mouthrinse, matrix metalloproteinase-8 (including aMMP-8, neutrophil collagenase, PerioSafe, ImplantSafe, and ORALyzer), and calprotectin (including S100A8, S100A9, and MRP8/14). A fully specified query combining all five concept blocks returned few records, so we iteratively broadened the evidence base with narrower query combinations and backward and forward citation tracking of key papers and consensus documents [18,20]. We prioritized studies evaluating oral-rinse and mouthrinse aMMP-8 immunotesting in relation to periodontal disease activity and treatment monitoring; studies of oral-fluid calprotectin in relation to periodontal inflammation and systemic inflammatory burden; and studies linking oral inflammation to dysglycemia and cardiovascular risk relevant to referral pathways. We included methodological papers on pre-analytics and sampling standardization, along with selected mechanistic literature, to support biological plausibility. Where our group’s own exploratory analyses are mentioned or shown (Section 4.1), they are identified as such and treated as illustrative and hypothesis-generating; they are not used to support quantitative conclusions.

The search covered the period from database inception to 31 July 2026. The core Boolean strategy combined five concept blocks: (1) (periodontitis OR “periodontal disease*” OR “periodontal inflammation”); (2) (“diabetes mellitus, type 2”[MeSH] OR “type 2 diabetes” OR prediabetes OR dysglycemia OR “metabolic syndrome”); (3) (“point-of-care testing”[MeSH] OR “point of care” OR chairside OR “chair-side” OR “lateral flow”); (4) (“mouthrinse” OR “oral rinse” OR saliva OR “gingival crevicular fluid”); and (5) (“matrix metalloproteinase 8”[MeSH] OR “MMP-8” OR “aMMP-8” OR “neutrophil collagenase” OR calprotectin OR “S100A8” OR “S100A9” OR “MRP8/14”). Because the fully combined query returned few records, we combined blocks pairwise and in triplets, and performed backward and forward citation tracking of key papers and consensus documents as described above.

Eligibility criteria were as follows. We included peer-reviewed human clinical studies (diagnostic accuracy, cross-sectional, cohort, and interventional), systematic reviews and meta-analyses, consensus documents, and methodological papers on pre-analytics, published in English, that reported oral-fluid aMMP-8 or calprotectin (or both) in relation to periodontal disease activity, treatment monitoring, or cardiometabolic risk. We included mechanistic and in vitro studies only to support biological plausibility. We excluded case reports, conference abstracts without full text, animal-only studies, and studies of non-oral biofluids without relevance to the referral question. No restriction was placed on publication year. Titles and abstracts were screened, and full texts were selected and prioritized, by the first author, with verification by the senior authors; disagreements were resolved by discussion. Studies were prioritized when they reported oral-rinse or mouthrinse data, a chair-side test format, longitudinal treatment monitoring, or a direct link to dysglycemia or cardiovascular risk.

## 3. Biological Rationale

### 3.1. Neutrophil-Driven Inflammation at the Periodontitis–Cardiometabolic Interface

At the cellular level, neutrophils, monocyte subsets, and resident immune cells release proteolytic enzymes (notably matrix metalloproteinases), reactive oxygen species, and cytokines that can promote repair or, when chronically activated, sustain extracellular matrix breakdown and tissue injury. Hyperglycemia, dyslipidemia, and central adiposity amplify this response by stimulating endothelial and macrophage production of pro-inflammatory mediators, reinforcing a self-perpetuating loop at the metabolic–vascular interface [4,21,22]. Periodontitis fits this paradigm: driven by dysbiotic biofilms and a dysregulated host response, it generates a persistent neutrophilic infiltrate whose mediators, most notably aMMP-8 and calprotectin, reflect both collagenolytic tissue breakdown and innate inflammatory burden [8,20,23,24].

Bacterial proteases from the periodontal microbiota, including Treponema denticola chymotrypsin-like proteinase and Porphyromonas gingivalis gingipains, contribute by directly activating latent MMP-8 and MMP-9 in oral fluids [25].

Because the same effector pathways operate, at least in part, in cardiometabolic disease, oral-fluid biomarkers of neutrophil activation may carry information relevant beyond the periodontium; this is a mechanistic rationale, not a demonstrated clinical link.

### 3.2. Calprotectin (S100A8/A9)

Calprotectin is a heterodimer of the calcium-binding proteins S100A8 and S100A9 (also termed MRP8 and MRP14, or calgranulin A and B), expressed mainly by neutrophils, monocytes, and keratinocytes [10,26]. It is a validated fecal and serum marker of inflammation in conditions such as Crohn’s disease, cystic fibrosis, rheumatoid arthritis, and psoriasis [27,28,29,30]. Salivary calprotectin is elevated in several oral and systemic conditions, including gingivitis and periodontitis, geographic tongue, oral candidiasis, recurrent aphthous stomatitis, oral cancer, Sjögren syndrome, systemic lupus erythematosus, Behçet disease, and type 1 diabetes [31,32,33,34,35,36,37,38,39], and has more recently been evaluated in early-onset inflammatory bowel disease [40]. In periodontal contexts, calprotectin is elevated in serum, GCF, and saliva in periodontitis and in periodontitis with diabetes, and decreases after initial periodontal therapy [41,42,43,44].

Reported relationships with glycemic status are informative but not uniform. Salivary calprotectin has correlated with glycemic measures in some datasets, and GCF calprotectin has correlated with HbA1c [43,45]; however, the direction and strength of these associations vary across studies and time points, and some report reductions that track HbA1c improvement after therapy while others do not [43,46].

### 3.3. The Calprotectin–MMP-8 Zinc Axis and the Basis for Combined Interpretation

**Figure 1:**
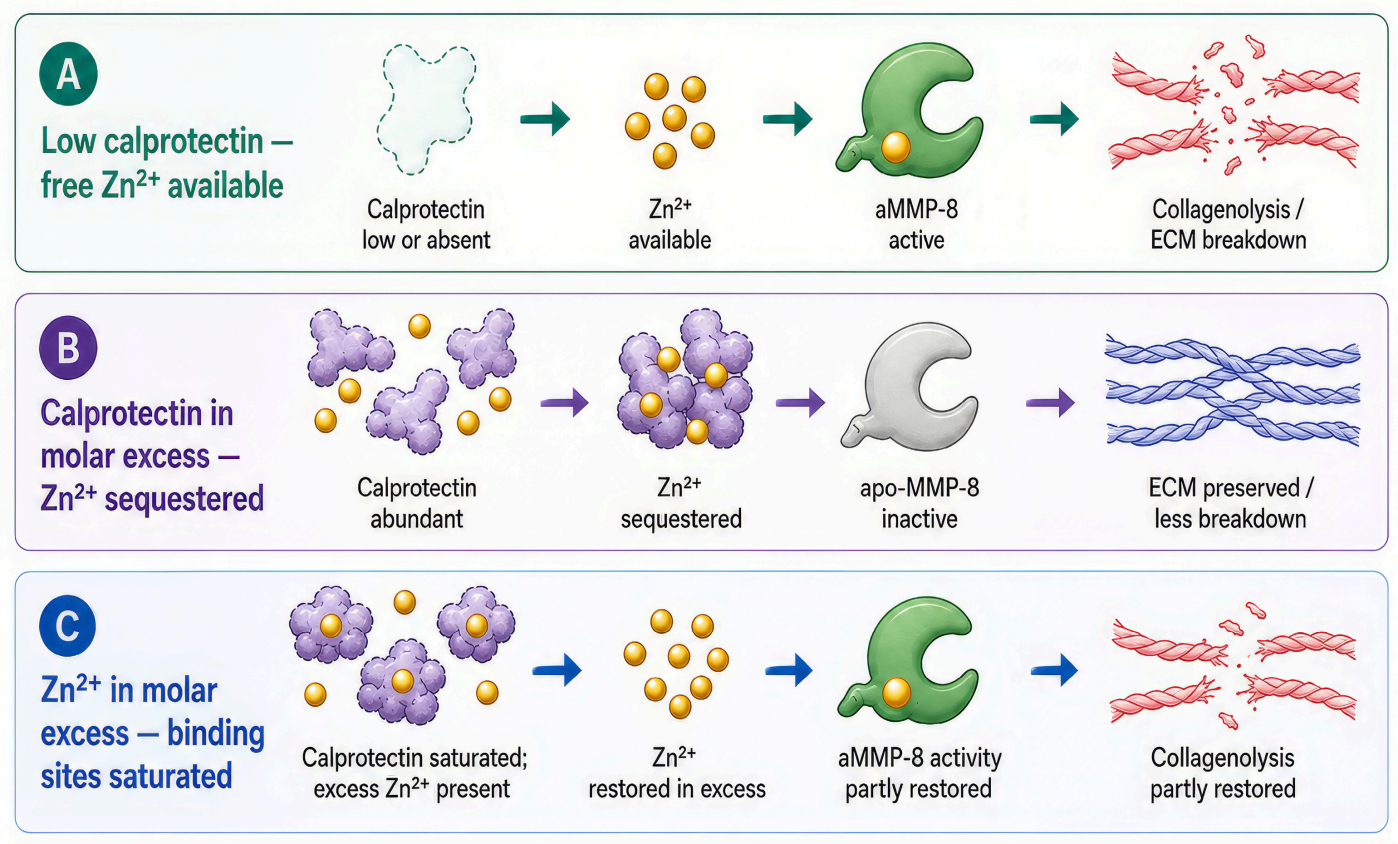
**Conceptual calprotectin-MMP-8 zinc axis.** The schematic illustrates the proposed modulation of active matrix metalloproteinase-8 (aMMP-8) by calprotectin through zinc (Zn^2+^) sequestration. (**A**) When calprotectin is low or absent, Zn^2+^ remains available to support aMMP-8 activity, promoting collagenolysis and extracellular matrix (ECM) breakdown. (**B**) When calprotectin is present in molar excess, it sequesters Zn^2+^, reducing the availability of the catalytic metal and thereby inhibiting aMMP-8 activity (the zinc-free apo-MMP-8 form), with relative preservation of the ECM. (**C**) When Zn^2+^ is present in excess and calprotectin-binding sites become saturated, Zn^2+^ availability and aMMP-8 activity may be partially restored, resulting in renewed collagenolysis. This mechanism is biologically plausible but has not yet been verified in oral fluids. The schematic is based on Isaksen and Fagerhol [11] and is an original illustration by the authors. The authors prepared the schematic with the assistance of AI-based illustration tools and reviewed and approved it (see the Use of Artificial Intelligence statement). Abbreviations: aMMP-8, active matrix metalloproteinase-8; ECM, extracellular matrix; MMP, matrix metalloproteinase; Zn^2+^, zinc ion.

Beyond its antimicrobial role, calprotectin can act as an endogenous modulator of MMP activity. Isaksen and Fagerhol reported that calprotectin inhibits MMP-2, -3, -7, -8, -9, and -13 in a concentration-dependent, enzyme-selective manner, with MMP-8 among the most sensitive; inhibition was relieved when zinc was supplied in molar excess, identifying sequestration of the catalytic metal, rather than active-site binding, as the mechanism [11] (Figure 1). This offers a conceptual reason why aMMP-8 and calprotectin could be complementary rather than redundant: chair-side aMMP-8 immunoassays detect an epitope of the activated enzyme and do not measure catalytic turnover, whereas calprotectin may constrain the activity of that same enzyme pool by limiting zinc availability [9].

This rationale should be regarded as plausible rather than demonstrated. The observations derive from a cell-free system using micromolar zinc, whereas free zinc in vivo is buffered in the picomolar-to-nanomolar range [12]; the extent to which calprotectin restrains collagenolysis in the periodontal pocket has not been established, and the mechanism has not been verified in oral fluids. Accordingly, the case for combined interpretation in this review rests on complementary clinical information rather than on a proven in vivo interaction.

## 4. Evidence Synthesis

### 4.1. Single-Marker Versus Combined-Marker Performance

Across oral-fluid studies, both aMMP-8 and calprotectin are higher in periodontitis than in health, but their diagnostic performance differs markedly. Oral-rinse aMMP-8 shows the more robust single-marker discrimination, whereas calprotectin alone is a weaker discriminator despite its relevance to neutrophil activation [43,47]. A salivary biomarker panel identified by Ramseier et al. associated MMP-8, MMP-9, and calprotectin with disease status, with a stronger association for the MMP markers than for calprotectin [48,49,50,51].

Two recent observations bear directly on complementarity. First, in hospitalized neurogeriatric inpatients with minimal systemic inflammation, GCF aMMP-8 and calprotectin were positively correlated, and each correlated with clinical parameters; importantly, neither correlated with C-reactive protein or white cell count, so in that population both behaved as predominantly local periodontal signals rather than systemic markers [52]. This finding tempers any claim that oral calprotectin *mirrors* systemic inflammatory burden; on current evidence, its systemic relevance is a hypothesis, not an established property. Second, complementary receptor-based signaling (elevated salivary AGE and RAGE with reduced sRAGE in uncontrolled T2D with periodontitis) provides additional mechanistic context for the diabetes–periodontitis interface [53,54].

**Figure 2:**
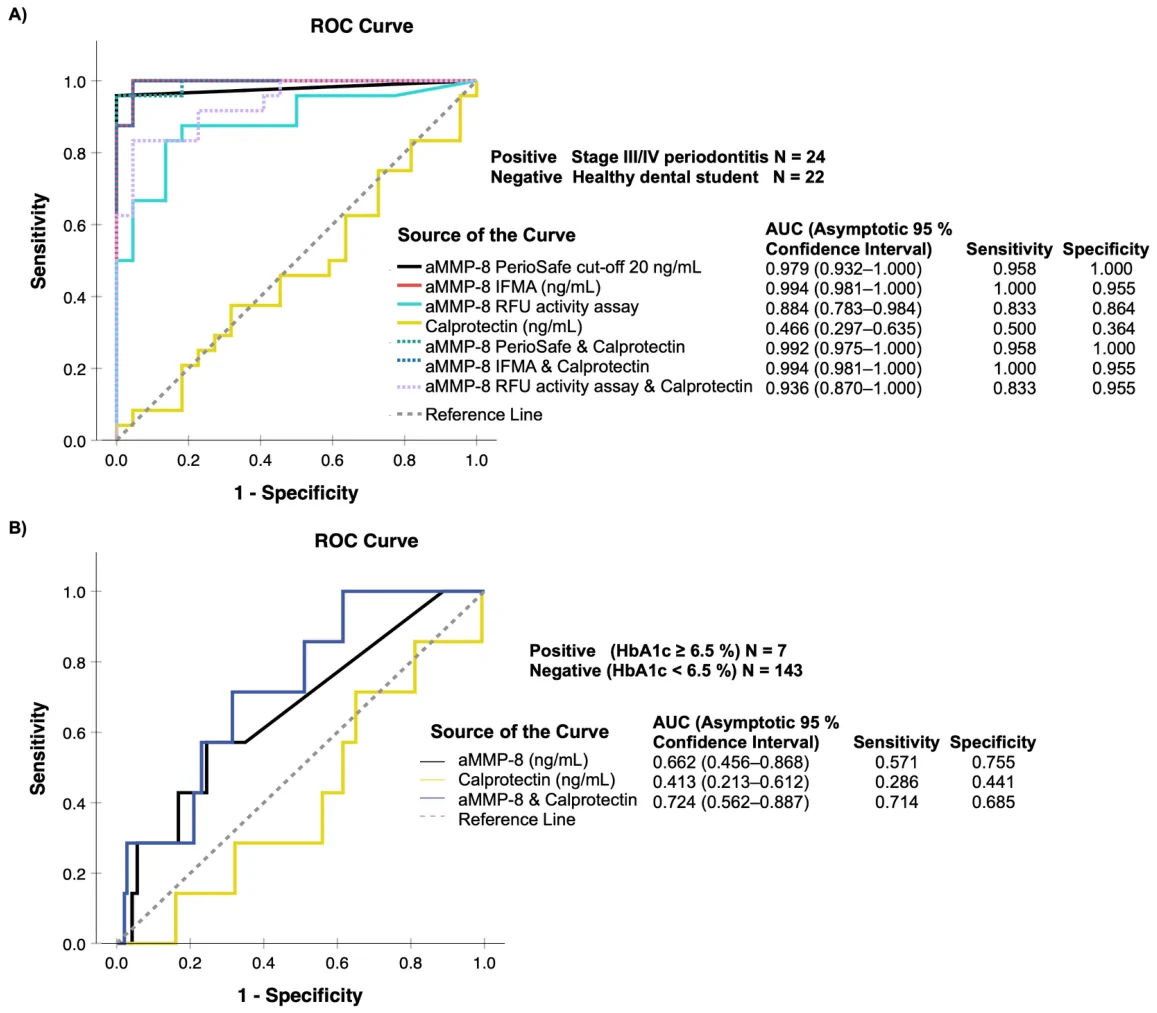
Exploratory ROC analyses of aMMP-8 and calprotectin, alone and combined (logistic regression), previously presented in our group’s special report [17]. (**A**) ROC analysis among 24 Finnish patients with stage III/IV, grade B/C periodontitis attending the Helsinki University Hospital Oral and Maxillofacial Diseases Clinic and 22 Finnish dental students from the University of Helsinki serving as periodontally and systemically healthy controls, using aMMP-8 and calprotectin independently and as a combination variable calculated by logistic regression; patient characteristics are as described previously [55,56]. The analysis suggests that the combination of aMMP-8 and calprotectin could be a potential biomarker pair for identifying patients with periodontitis. We present each variable’s area under the curve (AUC, with asymptotic 95 percent confidence interval) and the optimal sensitivity and specificity calculated by Youden’s index. (**B**) ROC analysis using aMMP-8 and calprotectin independently and as a combination variable (logistic regression) for identifying patients with HbA1c at or above 6.5 percent (*N* = 150 Greek patients, of whom seven were positive; patient characteristics as described previously [14]). We present each variable’s AUC and the optimal sensitivity and specificity calculated by Youden’s index. These analyses are illustrative and hypothesis-generating and do not form part of this review’s evidence synthesis. Abbreviations: aMMP-8, active matrix metalloproteinase-8; AUC, area under the curve; HbA1c, glycated hemoglobin; IFMA, immunofluorometric assay; RFU, relative fluorescence units; ROC, receiver operating characteristic. All ethical approvals and informed consent procedures for these cohorts are reported in the Institutional Review Board and Informed Consent Statements.

**Exploratory observations (illustrative, hypothesis-generating).** Exploratory analyses by our group, using conventional multivariable logistic regression, are consistent with the hypothesis that combining aMMP-8 and calprotectin improves discrimination relative to either marker alone, both for advanced periodontitis and, in a separate sample, among patients with HbA1c at or above 6.5 percent. These analyses were previously presented as illustrative, hypothesis-generating data in our group’s special report [17], derive from single, non-representative samples, and are hypothesis-generating; they are presented here as illustrative only and are not used to support quantitative conclusions. The corresponding receiver operating characteristic (ROC) analyses are shown in Figure 2; in the periodontitis sample (24 Finnish patients with stage III/IV, grade B/C periodontitis versus 22 periodontally and systemically healthy dental students), the combined aMMP-8 and calprotectin variable reached areas under the curve (AUCs) of 0.936 to 0.994 depending on the aMMP-8 assay format, whereas calprotectin alone reached 0.466; in the dysglycemia sample (150 Greek patients, of whom seven had HbA1c at or above 6.5 percent), the combination reached an AUC of 0.724 versus 0.662 for aMMP-8 and 0.413 for calprotectin alone [14,17,55,56]. These observations remain deliberately excluded from the evidence synthesis and from Table 1, and no clinical inference should be drawn from them. They are shown solely for transparency, particularly in view of the patent interests declared in the Conflicts of Interest statement, and definitive conclusions will require prospective validation in representative cohorts.

Taken together, the evidence supports oral-rinse aMMP-8 as the stronger single marker of periodontal disease activity and supports combined interpretation as a promising but not yet validated refinement. A dual-marker POCT strategy is therefore best framed as a risk-stratification adjunct to conventional periodontal assessment and as a potential trigger for interdisciplinary referral rather than as a stand-alone diagnostic substitute [49]. Table 1 summarizes the principal clinical studies underpinning this synthesis, stratified by population, oral-fluid matrix, assay format, cut-off, outcome, and validation status.

### 4.2. Pre-Analytical Considerations

Oral rinse, saliva, and GCF are not interchangeable matrices. A direct comparison across all three in the same subjects found that biomarker concentrations and their discriminative behavior differ by sampling site, so thresholds derived in one matrix cannot be transferred to another [63]. The same applies to assay format: aMMP-8 measured in the same samples by laboratory immunoassay and by chair-side test are correlated but not equivalent, and the chair-side format has its own detection limit and dynamic range [64]. aMMP-8 has been characterized most extensively in GCF [65,66], with mouthrinse emerging later as a pragmatic chair-side option [20]; mouthrinse collagenase measurement is not new, effectively collecting GCF from all teeth at once, and has been described as early as 1990 [67]. What has changed is not the analyte but the assay’s accessibility. The practical corollary is that a threshold is a property of a matrix assay–population combination, not of the biomarker, a point that directly conditions the interpretation of the cut-off values discussed below.

Reported diagnostic performance of aMMP-8 point-of-care tests varies substantially with matrix, threshold, and population. In the developers’ work, a 20 ng/mL cut-off has been proposed as optimal for distinguishing periodontal health from disease and for staging and grading, with sensitivity and specificity commonly in the range of roughly 70 to 90 percent [9,68]. Independent diagnostic-accuracy studies and meta-analyses have reported lower and more variable performance, particularly lower sensitivity [58,59,60,69]. In these analyses, raising the cut-off traded sensitivity for specificity, and neither the threshold nor the oral-fluid type significantly changed overall accuracy, so the disagreement concerns the level of discrimination achievable rather than the superiority of any single cut-off. These differences reflect genuine methodological heterogeneity (matrix, sampling protocol, case definition, and population) rather than a settled disagreement, and they should be presented to readers as an open question. At the same time, a substantial body of independent and multi-population evidence supports 20 ng/mL as the preferred operating point: comparative analyses in Finnish, Indian, and Turkish cohorts have repeatedly favored 20 ng/mL over the 10 and 25 ng/mL alternatives as the more conservative choice [70,71,72,73], and both Matthews-correlation-based cut-off optimization and AI-assisted polynomial modeling of mouthrinse data have independently identified 20 ng/mL as optimal for detecting the presence and stage of periodontitis [68,74,75].

### 4.3. Treatment Monitoring

A chair-side biomarker is clinically useful only if it changes with treatment. Oral-rinse aMMP-8 POCT identifies patients with a high periodontal inflammatory burden [57], and aMMP-8 concentrations decrease after non-surgical periodontal therapy [70,76]. In GCF, MMP-8 response patterns rather than single measurements predict site-level treatment outcome [77], and chair-side aMMP-8 tracked treatment response over 24 weeks in stage III/IV periodontitis [61]. Two caveats apply: reported follow-up intervals range from about 6 to 24 weeks, and the interval at which a persistently elevated result should prompt re-intervention is not established; and the operating characteristics of cut-offs validated for case identification have not been shown to hold during monitoring.

Monitoring data for oral-fluid calprotectin are considerably sparser than for aMMP-8, and no threshold for treatment response has been proposed. This asymmetry matters for any dual-biomarker workflow: the two markers are not equally mature as monitoring tools, and a combined protocol should not assume that calprotectin can be interpreted longitudinally in the way aMMP-8 can [78].

### 4.4. The Periodontitis–Cardiometabolic Interface

Longitudinal evidence supports a bidirectional temporal association between periodontitis and type 2 diabetes [79], within a broader umbrella of established links between oral health and multiple systemic noncommunicable diseases [80]. Periodontal inflammation is linked to metabolic syndrome (MetS) and T2D through shared pathways involving neutrophil activation, oxidative stress, insulin resistance, dysregulated lipid metabolism, and low-grade systemic inflammation [4,22,53,54,79,81,82,83,84,85,86,87]. In MetS-associated periodontitis, greater periodontal destruction and higher oral-fluid aMMP-8 and myeloperoxidase have been reported alongside worse periodontal indices, whereas total MMP-8 is less informative for disease activity [70,71]. A high proportion of positive aMMP-8 point-of-care results in MetS-associated periodontitis, contrasted with their absence in periodontally healthy controls, supports the utility of oral-rinse aMMP-8 for identifying active disease [68].

Chair-side aMMP-8 testing has been proposed as an opportunistic route to prediabetes and diabetes screening in the dental setting, on the rationale that dental attendance may precede medical diagnosis [14,15,16], and prediabetes has been associated with MMP-8 activation and accelerated periodontal destruction [86,88]. Additional salivary markers (for example, interleukin-17, developmental endothelial locus-1, and various proteomic and microbiome signatures) have been examined in periodontitis with T2D, reinforcing that oral fluids carry cardiometabolically relevant information, though none is yet a validated chair-side test [89,90,91,92]. These observations are compatible with bidirectional interactions between periodontal and cardiometabolic dysregulation and motivate oral-fluid testing as a pragmatic, non-invasive adjunct for identifying patients who may benefit from cardiometabolic assessment, while stopping short of demonstrating that biomarker-triggered referral improves outcomes. This relationship is bidirectional. CVD patients—as well as DM patients [13,93]—may benefit from aMMP-8 POCT screening in medical settings to identify their risk of periodontitis, enabling referral to a dentist for oral health examination and, if necessary, appropriate treatment [14].

## 5. Discussion

### 5.1. Clinical Implications and What This Review Adds

The combined use of oral-fluid aMMP-8 and calprotectin is a biologically plausible dual-biomarker strategy that targets local collagenolytic activity and neutrophil-driven inflammation. Interpreted together within standardized chair-side workflows, the two markers might enhance risk stratification for periodontitis and, potentially, for associated cardiometabolic conditions [17,94]. The contribution of the present review is deliberately cautious. Compared with the recent proposal to combine aMMP-8, calprotectin, and CCAA imaging as an integrated tool [17], we do not argue that such a tool is ready for use; we argue that its individual components are at very different stages of validation, that oral calprotectin is the least mature of them, and that the pre-analytical and threshold problems described in Section 4.2 must be solved before combination can be more than hypothesis-generating. Framed this way, dual-marker testing serves as a structured prompt for clinical attention and referral, not a diagnostic substitute. Used in tandem as a unique complementary pair, aMMP-8 and calprotectin may extend chair-side risk stratification not only within dental settings but also toward medical referral pathways at the periodontitis–cardiometabolic interface.

This framing also resolves an apparent tension in the literature. Oral calprotectin has been described both as a marker of systemic inflammatory burden and as a predominantly local periodontal signal. The available oral-fluid data, including the finding that calprotectin did not correlate with systemic inflammatory markers in a low-inflammation population [52], favor the local interpretation. Its systemic and cardiometabolic relevance remains a reasonable hypothesis to test, not a property to assume.

The broader oral-fluid biomarker landscape supports and extends this framing. At the biological interface between periodontitis and diabetes, cytokine and growth-factor profiling of platelet-rich fibrin and hyperacute serum from periodontitis patients with and without T2DM has demonstrated a measurably altered inflammatory milieu in the coexisting-disease state [95], reinforcing the mechanistic rationale outlined in Section 3. Beyond MMP-8 and calprotectin, salivary sclerostin (SOST), Dickkopf-related protein 1 (DKK1), and WNT5a have recently shown high discriminative performance (areas under the curve above 0.90) for detecting periodontitis and for separating grade B from grade C disease [96], illustrating that grading-relevant information is recoverable from saliva, although these markers likewise await chair-side translation and external validation. At the cardiovascular interface, serum paraoxonase-1 activity was significantly lower in ischemic heart disease patients with stage III/IV periodontitis than in cardiac patients with a healthy periodontium [97], supporting the inclusion of oxidative and atheroprotective mechanisms in the periodontal–cardiometabolic interface that dual-biomarker testing aims to flag. Together, these studies broaden the biomarker context of this review and underline that aMMP-8 and calprotectin are two members of a wider candidate panel whose comparative and combined performance remains to be established.

### 5.2. Toward Integrated Chair-Side Screening: Imaging, Workflow, and AI

**Figure 3:**
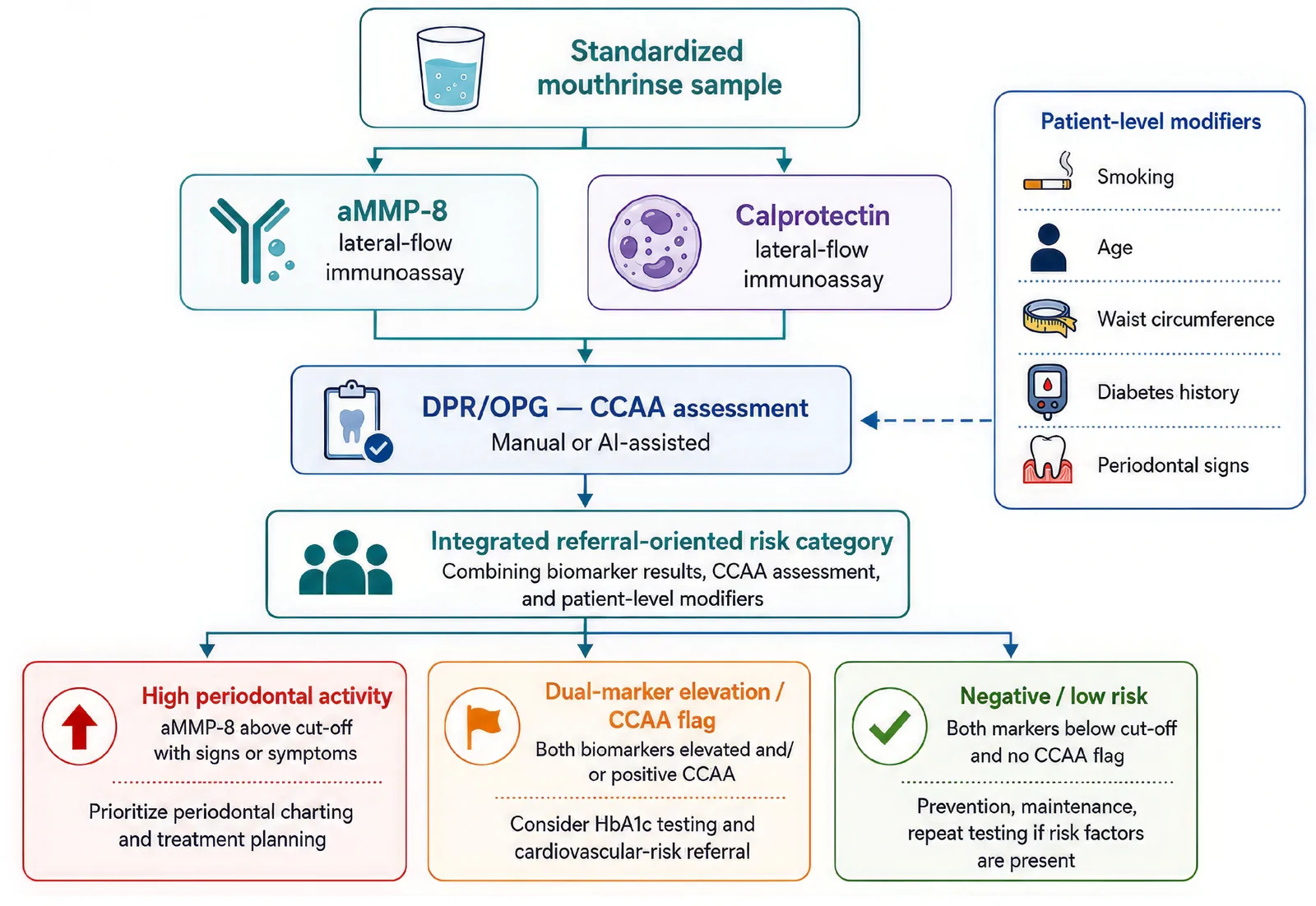
**Proposed chair-side decision pathway for dual-biomarker screening.** A standardized mouthrinse sample is analyzed using lateral-flow immunoassays for active matrix metalloproteinase-8 (aMMP-8) and calprotectin. Biomarker results are interpreted together with panoramic radiographic assessment (manual or AI-assisted) for calcified carotid artery atheroma (CCAA) and patient-level modifiers, including smoking, age, waist circumference, diabetes history, and periodontal signs, to assign an integrated referral-oriented risk category. Patients with **high periodontal activity** (aMMP-8 above the cut-off with compatible signs or symptoms) should undergo comprehensive periodontal evaluation and treatment planning. Patients with **dual-marker elevation and/or a positive CCAA flag** may be considered for HbA1c testing and cardiovascular-risk referral. Patients classified as **negative/low risk** may continue preventive care, maintenance, and repeat testing when risk factors are present. This pathway is proposed as an adjunctive clinical workflow and has not yet been prospectively validated; figure adapted from the authors. The authors prepared the figure with the assistance of AI-based illustration tools and reviewed and approved it (see the Use of Artificial Intelligence statement). Abbreviations: AI, artificial intelligence; aMMP-8, active matrix metalloproteinase-8; CCAA, calcified carotid artery atheroma; HbA1c, glycated hemoglobin.

Beyond periodontal triage, oral-rinse biomarkers could in principle be combined with routinely acquired dental imaging. Calcified carotid artery atheromas (CCAAs) can be identified on standard dental panoramic radiographs and have been proposed as an opportunistic marker of subclinical atherosclerotic burden [98]; because the radiograph is already acquired for dental indications, this assessment adds neither cost nor radiation [99]. Integrating biomarker and imaging signals with patient-level modifiers (smoking, age, waist circumference, and diabetes history) could support earlier risk stratification and more targeted referral [17]. Table 2 and Figure 3 summarize a conceptual ten-minute chair-side workflow and referral triggers. We emphasize that this pathway is proposed and has not been prospectively validated; the dual-marker referral trigger is proposed rather than an established decision rule. Such combined interpretation of biomarker, imaging, and modifier inputs could, in future, be supported by AI technologies to standardize scoring and improve the consistency of referral decisions [100]. In this respect, Mai et al. reported that point-of-care assessment of CCAAs on routine dental radiographs can be performed with a sensitivity of approximately 80 percent and a specificity of approximately 98 percent, underscoring the public-health opportunity for cardiovascular risk reduction in dental settings [99].

Artificial intelligence may eventually improve the reproducibility of CCAA detection and support multivariable risk modeling that combines biomarkers, imaging, and clinical covariates [101,102]. At present, such pipelines should be considered adjunctive and require external validation, transparency, and explicit attention to false positives and referral burden. In the same spirit, a validated polynomial risk model (PERIORISK) integrates a mouthrinse aMMP-8 result (20 ng/mL cut-off), smoking status, the product of waist circumference and age, and personal or parental diabetes history [68,74]. PERIORISK is best viewed as an extension of chair-side aMMP-8 testing that embeds the result within a broader cardiometabolic context rather than as a stand-alone diagnostic. In the original modeling cohort, PERIORISK reached a sensitivity of approximately 94 percent, a specificity of approximately 74 percent, and an accuracy of approximately 90 percent [68,74]. Because these figures were derived and tested on the same dataset used to build the model, they represent apparent (in-sample) performance and require external validation before clinical adoption.

### 5.3. Limitations

This review is intentionally narrative and thematically selective; it does not provide pooled estimates or formal certainty grading. The underlying evidence base is heterogeneous in sampling protocols (oral rinse versus saliva versus GCF), assay formats (laboratory versus POCT), case definitions, and thresholds, which limits comparability and may inflate apparent performance in small, selected cohorts. Cardiometabolic endpoints are largely inferred from cross-sectional associations rather than adjudicated outcomes. Mechanistic evidence for the calprotectin–MMP axis comes from cell-free systems at non-physiological zinc concentrations and has not been verified in oral fluids [11,12]. Several authors hold patents relating to aMMP-8 lateral-flow testing (see Conflicts of Interest), and we have therefore made a deliberate effort to present independent and less favorable evidence alongside the developers’ data [58,59,60,69]. In addition, no population-based reference ranges and no industry-wide consensus cut-off values currently exist for either marker in oral rinse; this review therefore makes no recommendation for clinical adoption and positions dual-marker testing strictly as a research-stage, hypothesis-generating adjunct pending the validation steps outlined in Section 5.4.

### 5.4. Future Research

Four conditions should be satisfied before a combined workflow could be recommended for practice. First, prospective validation in representative populations against the 2017 case definitions, with thresholds fixed in advance, a step not yet completed for any combined algorithm [68,74]. Second, resolution of the discrepant estimates of aMMP-8 diagnostic accuracy and of the unsettled relative performance of the two markers [59,60]. Third, establishment of age- and care-setting-specific reference values for oral-fluid calprotectin, which do not presently exist. Automated CCAA detection on panoramic radiographs is a plausible route to scale [101,102], but whether such tools improve identification of at-risk patients across the periodontitis–dysglycemia–cardiovascular axis, rather than automate existing uncertainty, must be tested rather than assumed. Fourth, population-based screening studies in unselected cohorts are needed to establish reference distributions for both markers, and AI-assisted risk models that extend validated aMMP-8-based algorithms such as PERIORISK by incorporating calprotectin as an additional input should be developed and then externally validated rather than assumed to perform; such modeling requires prospective patient-level datasets that do not yet exist and is therefore beyond the scope of a narrative review [58,59,60,69].

## 6. Conclusions

Oral-rinse point-of-care testing for aMMP-8 and calprotectin is a promising adjunct for periodontal assessment at the periodontitis–cardiometabolic interface. The two markers are biologically complementary in principle: aMMP-8 reflects active collagenolytic tissue breakdown, while calprotectin reflects neutrophil-driven inflammation whose systemic relevance, on current oral-fluid evidence, remains a hypothesis rather than an established property. Interpreted together within standardized workflows, combined information may support identification of patients with active periodontal disease and appropriate referral for cardiometabolic assessment. However, the evidence remains heterogeneous with respect to sampling, assay format, case definitions, and thresholds; oral-fluid calprotectin lacks validated reference ranges; and no combined algorithm has been prospectively validated. Standardized pre-analytics, transparent threshold reporting, reference values for oral calprotectin, and prospective multicenter validation, together with realistic evaluation of AI-assisted models, are prerequisites for clinical implementation. Positioned honestly as an adjunct to, not a substitute for, conventional periodontal examination and interdisciplinary care, dual-biomarker chair-side testing is a reasonable direction for further research at the intersection of oral and systemic health.

## 7. Patents

The aMMP-8 lateral-flow point-of-care technology discussed in this review is covered by patents held by T.S., listed under Conflicts of Interest.

## Figures and Tables

**Table 1:** Summary of principal clinical studies of oral-fluid aMMP-8 and calprotectin, by population, matrix, assay, cut-off, outcome, and validation status. Matrices are not interchangeable; thresholds are properties of a specific matrix assay–population combination and cannot be transferred across settings.

Study [ref]	Population/Setting	Matrix	Assay	Cut-Off	Outcome (Diagnostic/Monitoring)	Validation Status
Sorsa et al., 2020 [9]	Adults; periodontitis staging and grading cohorts	Mouthrinse	aMMP-8 lateral-flow POCT with digital reader	20 ng/mL	Diagnostic; adjunct to staging and grading	Developer-derived; in-sample performance; no external validation
Leppilahti et al., 2011 [57]	Adults with periodontal inflammatory burden	Oral rinse	aMMP-8 POC immunotest	Qualitative	Diagnostic; identifies high inflammatory burden	Single cohort; exploratory
Deng et al., 2021 [58]	Adults; periodontal health versus disease	Mouthrinse	aMMP-8 POCT	20 ng/mL and alternatives evaluated	Diagnostic accuracy	Independent evaluation; lower sensitivity than developer reports
Wei et al., 2025 [59]	Adults (pooled populations)	Saliva	aMMP-8 POCT	Various	Diagnostic accuracy (systematic review and meta-analysis)	Independent synthesis; variable performance
Li et al. [60]	Adults; periodontal health versus disease	Oral rinse	aMMP-8 POCT	Various	Diagnostic accuracy	Independent evaluation
Yilmaz et al. [61]	Stage III/IV periodontitis; 24-week follow-up	Mouthrinse	aMMP-8 POCT	20 ng/mL	Monitoring; tracked treatment response	Single-center; cut-off not validated for monitoring
Rintamarttunen et al. [62]	Periodontitis patients after non-surgical therapy	Mouthrinse	aMMP-8 POCT	20 ng/mL	Monitoring; concentrations decrease after therapy	Small cohorts; monitoring thresholds not validated
Grigoriadis et al., 2021 [14]	Dental patients screened for dysglycemia	Mouthrinse	aMMP-8 POCT	20 ng/mL	Referral; prediabetes/diabetes screening signal	Exploratory; single setting
Kido et al. [47]	Periodontitis patients; site-level diagnosis	GCF	Immunochromatographic calprotectin assay	Assay-specific	Diagnostic; identification of diseased sites	Single population; no oral-fluid reference ranges
Kim et al., 2020 [44]	Adults; periodontitis versus health	Saliva	Laboratory immunoassays (MMP-9, S100A8)	Study-derived	Diagnostic and prognostic modeling	Derivation only; no external validation
Haririan et al. [42]	Periodontitis versus periodontal health	Saliva and serum	Laboratory immunoassay (calprotectin)	None proposed	Diagnostic association	Cross-sectional; no thresholds
Gao et al. [43]	Periodontitis with and without T2DM	GCF and serum	Laboratory immunoassay (calprotectin)	None proposed	Monitoring; decrease after initial therapy	Small samples; no validated thresholds
Meyer-Hofmann et al., 2026 [52]	Neurogeriatric inpatients with minimal systemic inflammation	GCF	Laboratory assays (aMMP-8 and calprotectin)	None	Both markers behaved as local periodontal signals	Hypothesis-generating
Räisänen et al., 2025 [17]	Conceptual special report	Mouthrinse plus panoramic radiograph	aMMP-8 and calprotectin POCT plus CCAA assessment	20 ng/mL (aMMP-8)	Proposed integrated risk assessment and referral	Not prospectively validated

*Abbreviations: aMMP-8, active matrix metalloproteinase-8; CCAA, calcified carotid artery atheroma; GCF, gingival crevicular fluid; MMP, matrix metalloproteinase; POC(T), point-of-care (testing); T2DM, type 2 diabetes mellitus.*

**Table 2:** Conceptual ten-minute chair-side dual-biomarker workflow and referral triggers. This workflow is conceptual and has not been prospectively validated; timings are indicative, cut-offs are assay- and manufacturer-dependent, and referral pathways should follow local clinical governance.

Time (min:s)	Procedure	Documentation
00:00-01:00	Collect a standardized mouthrinse sample (defined volume, defined rinse time).	Recent food intake, smoking, gingival bleeding, current antibiotic therapy.
01:00–06:00	Perform lateral-flow immunoassays for aMMP-8 and calprotectin per manufacturer instructions.	Qualitative result and/or digital reader value (ng/mL); cut-off applied.
06:00–08:00	Review the panoramic radiograph (DPR/OPG) for CCAA using a structured checklist.	Checklist outcome: automated or AI-assisted output and confidence score, where available.
08:00–10:00	Integrate biomarker and radiographic findings with modifiers: smoking, age, waist circumference, diabetes history, periodontal signs.	Assigned referral-oriented risk category (see triggers below).
**Risk profile**	**Trigger findings**	**Recommended action**
High periodontal activity	aMMP-8 above cut-off, with periodontal symptoms or signs.	Prioritize comprehensive periodontal charting and treatment planning.
Dual-marker elevation	aMMP-8 and calprotectin both elevated, and/or a positive CCAA flag.	Consider timely medical referral for glycemic assessment (HbA1c) and cardiovascular risk evaluation, consistent with local pathways.
Negative/low risk	Both markers below cut-off; no CCAA flag.	Reinforce prevention and periodontal maintenance; repeat testing at defined intervals if risk factors are present.

*Abbreviations: aMMP-8, active matrix metalloproteinase-8; CCAA, calcified carotid artery atheroma; DPR/OPG, dental panoramic radiograph (orthopantomogram); HbA1c, glycated hemoglobin.*

## Data Availability

The datasets analyzed in Figure 2 were derived from previously published studies [14,17,55,56], whose full descriptions and data availability are reported in the original publications. No additional new data were generated for the present narrative review.
